# Bio-inspired circular latent spaces to estimate objects' rotations

**DOI:** 10.3389/fncom.2023.1268116

**Published:** 2023-11-24

**Authors:** Alice Plebe, Mauro Da Lio

**Affiliations:** Department of Industrial Engineering, University of Trento, Trento, Italy

**Keywords:** neuro-inspired artificial intelligence, robotic grasping, rotation detection, bio-inspired neural networks, ellipsoid body

## Abstract

This paper proposes a neural network model that estimates the rotation angle of unknown objects from RGB images using an approach inspired by biological neural circuits. The proposed model embeds the understanding of rotational transformations into its architecture, in a way inspired by how rotation is represented in the ellipsoid body of *Drosophila*. To effectively capture the cyclic nature of rotation, the network's latent space is structured in a circular manner. The rotation operator acts as a shift in the circular latent space's units, establishing a direct correspondence between shifts in the latent space and angular rotations of the object in the world space. Our model accurately estimates the difference in rotation between two views of an object, even for categories of objects that it has never seen before. In addition, our model outperforms three state-of-the-art convolutional networks commonly used as the backbone for vision-based models in robotics.

## 1 Introduction

Accurately measuring the angles of rotation of objects is essential for a wide range of applications, including robotics. As the complexity of these applications increases, artificial vision has become an indispensable tool (Du et al., 2021; Yin and Li, 2022). In this context, deep convolutional neural networks have emerged as the preferred choice (Caldera et al., 2018; Tian et al., 2023). However, these methods use neural networks to learn from scratch how the physical transformation of rotation works. Using these methods in applications where rotation sensitivity is essential—as in most robotics applications—can be inefficient. A more effective approach would be to develop a neural model that embeds the rotation operator in its inner architecture, without having to learn it from visual data. Such an approach would enable precise rotation estimation for objects across different categories.

Incorporating prior knowledge into deep neural networks is not straightforward, as it goes against the data-driven nature of artificial learning. However, there are techniques to embed prior knowledge by imposing particular architectures in the network. One potential way is by drawing inspiration from the neural structures of biological organisms.

In the natural world, living organisms possess innate knowledge that is not acquired through external experiences. This innate knowledge is made possible by specific structures encoded in their biological makeup, which are predetermined and stored in their genetic code. These specialized structures allow them to perform essential functions, behaviors, or instincts without the need for direct learning or prior exposure to the outside environment.

This work aims to develop a deep neural network that embeds the understanding of rotational transformations into its architecture. This embedding is inspired by how rotation is represented in the biological neural structures of some living organisms.

All animals possess, to some extent, an innate understanding of how rotation transforms objects through their sense of sight. In higher animals like mammals, this inherent understanding becomes intricately intertwined with other processes that extract more complex information within the visual cortex (DeYoe and Van Essen, 1988; Gulyas et al., 1993). As a result, isolating a single mechanism or neural structure solely dedicated to encoding rotations remains impossible. On the other hand, there are simpler organisms, such as insects, whose nervous system has been extensively studied, and their neural circuits are well-defined and easier to analyze. Insects may not be concerned with estimating the rotation of objects optimally with respect to a certain reference axis. However, one thing they are inevitably interested in is a way to know their own rotation relative to the external world. This awareness is essential to maintain a sense of direction and location while moving for a planned action: searching for food and water, avoiding prey, finding mates.

In the brain of the fruit fly *Drosophila*, independent studies have uncovered a unique method of representing heading rotations using a ring-like neural circuit (Green et al., 2017; Turner-Evans et al., 2017). Considering that rotation is a cyclic operation, a neural circuit for encoding rotations should also possess a circular structure. In other words, the neural representation should return to its initial state after undergoing incremental modifications by a certain angular step and completing a full rotation. Remarkably, the neural circuit of the *Drosophila* is circular because the neurons physically form a morphological ring.

We propose an autoencoder-like deep neural model featuring a “circular latent space” inspired by the ring-like neural circuit found in the *Drosophila*'s brain. Instead of estimating the insect's heading direction, our model is designed to estimate the angle of rotation between two views of an object. To enforce a cyclic structure within the latent representation, we incorporate a fixed operator that is linearly isomorphic to rotation and affects the circular latent space by cyclically shifting its units. This design creates a direct correspondence between a shift in the latent space and an angular rotation in the world. In practice, the model learns to map shifts in the latent space to rotations of an object, allowing it to estimate the rotation angle between two views of the same object. This approach enables the network to predict and estimate the rotations of previously unseen objects. We assume a single axis of rotation in this study but plan to extend this idea to more complex transformations in future work.

We evaluate the performance of our model on two datasets: one with planar rotations and the other with objects rotating in 3D space. Furthermore, we compare our model to three state-of-the-art convolutional networks that are used as the backbone of vision-based models for various robotic applications, and we show that our model outperforms the benchmarks.

The rest of the paper is organized as follows. Section 2 describes the brain structures that served as inspiration for our proposed model in representing rotations. Section 3 provides an overview of existing approaches utilizing deep convolutional networks in tasks requiring rotation estimation, along with a discussion of the challenges related to rotation invariance. Section 4 delves into the implementation of our model, elaborating on the circular latent space and the shift operator. In Section 5, we describe the datasets employed in this study and the preprocessing steps taken to ensure suitability for the rotation estimation task. Section 6 presents the results of our model on the two datasets, along with a comparison against benchmark models. Finally, Section 7 concludes the paper and discusses potential future developments.

The source code of the presented model is available at https://github.com/3lis/neuro-circ-latent.

## 2 Circular representations in the brain

Accurate estimation of a specific type of rotation is crucial for the daily behavior of most animals: the rotation of the animal's body relative to the environment. To be able to navigate the environment and reach a particular destination, the animal needs to know its own location in space and its spatial orientation represented by the body angle along the earth vertical axis (yaw). Neurons that encode this rotation information are referred to as *head direction* (HD) cells (Taube, 2007). These specialized neurons play a crucial role in providing animals with a sense of direction and orientation, enabling them to navigate accurately in their environment.

Several studies have detected HD cells in different brain regions, including the dorsal portion of the medial superior temporal area (MSTd) in monkeys (Takahashi et al., 2007). In this region, vision serves as the primary source for HD information, while secondary cues are derived from vestibular, proprioceptive, and motor inputs. However, investigating the coding mechanisms of HD cells in mammals presents a significant challenge due to their close connection with the visual system and their presence in areas comprising millions of neurons, making it impractical to study them extensively.

The limited number of neurons in insects has made them invaluable for unraveling the mechanisms of rotation encoding in the brain. A significant breakthrough in this area was achieved through two coinciding yet independent research studies: (Green et al., 2017) and (Turner-Evans et al., 2017). Both studies unveiled a fundamental neural circuit dedicated to encoding head directions in the fruit fly *Drosophila*. This essential neural circuit is located in the ellipsoid body, situated within the central complex of the fly's brain. Remarkably, the ellipsoid body comprises ~20,000 neurons, making it orders of magnitude simpler than similar structures in vertebrates.

**Figure 1:**
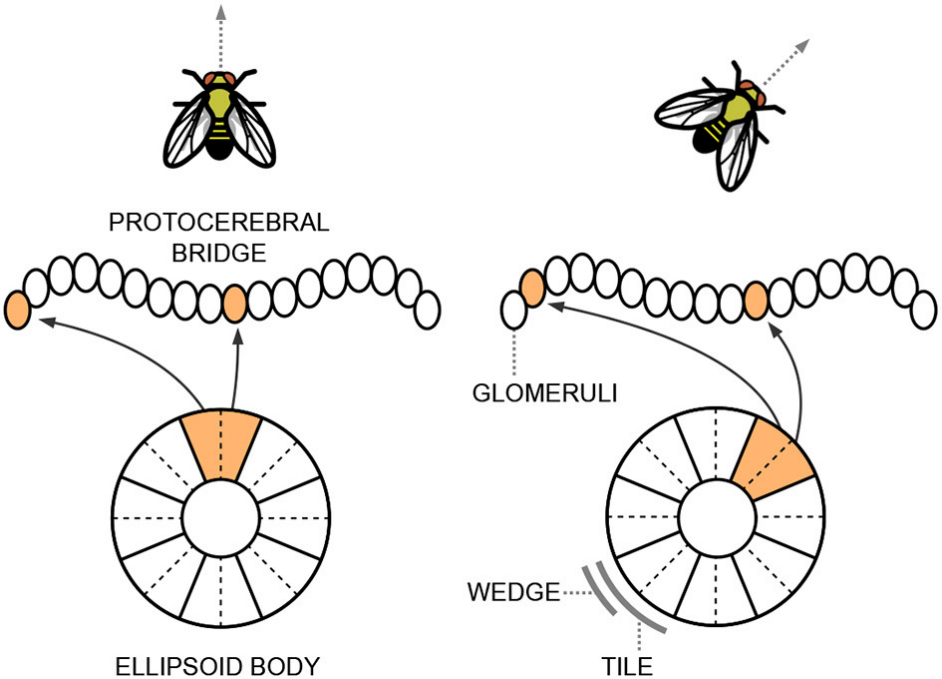
Simplified representation of the ellipsoid body and the protocerebral bridge situated within the central complex of the *Drosophila*'s brain. The neurons exhibit a bump of activity in a specific sector that corresponds to the current head direction of the fly.

As the name suggests, the ellipsoid body has a roughly circular structure and consists of eight sectors called “tile neurons”. Each tile is further divided into two “wedge neurons”. Figure 1 provides a simplified scheme of these structures. Tile and wedge neurons exhibit a bump of activity in a specific sector that corresponds to the current head direction, encoded from −π to π along the circular structure of the ellipsoid body. The tile neurons respond to changes in the animal's direction and trigger a shift in the activations of the wedge neurons. This continuous integration of information about body rotations updates the head direction representation. A shift of the bump of activity from one sector to a nearby one corresponds to a variation in orientation of ~ $\frac{\pi }{8}$ radians. Wedge neurons are strongly influenced by visual cues, while tile neurons are mainly activated by self-motion signals.

The ellipsoid body is primarily connected to another structure in the central complex, known as the protocerebral bridge, which resembles a handlebar in shape (see Figure 1). The protocerebral bridge consists of nine “glomeruli” in each hemisphere, arranged the one next to the other. The circular angle representation held in the ellipsoid body is linearly reflected in the left and right arms of the protocerebral bridge. It acts as a relay center connecting the left and right halves of the brain and plays a crucial role in fine-tuning motor outputs and integrating sensory information to regulate complex behaviors.

Building on the insights from these two seminal papers, researchers have conducted substantial work to decipher the details of the circular angular representation in the ellipsoid body. For example, they have explored how this representation is utilized to compute the allocentric traveling direction (Lyu et al., 2022).

While the level of detailed knowledge discovered in insects remains challenging to attain in vertebrates, a recent study by Petrucco et al. (2023) has uncovered a fascinating neural structure in zebrafish larvae that shares intriguing similarities with the ellipsoid body found in insects. In their research, they identified a population of 50–100 neurons in the anterior hindbrain responsible for encoding the heading direction of the animal. This neural population has been named r1π because the two principal components of their signals exhibit anticorrelation at an angle of π.

What makes this discovery even more remarkable is that the morphology of the r1π suggests a circular structure, corresponding to angles from −π to π. This circular arrangement bears striking similarities to the structure of the ellipsoid body observed in insects. Similar to how the ellipsoid body in *Drosophila* is associated with the linear structure of the protocerebral bridge, the r1π ring in zebrafish larvae is closely linked to a linear structure in the dorsal interpeduncular nucleus. This particular brain structure is known to play a role in spatial navigation and the generation of heading direction information.

These remarkable discoveries, demonstrating how organisms can encode innate knowledge within specific neural structures, hold intriguing implications for the development of novel algorithms for various robotic applications. In particular, our focus in this work is to harness the concept of representing rotation transformations using circular neural structures.

## 3 Rotation invariance in neural networks

The solutions presented in the previous Section, which address the problem of representing rotations in neural circuits of insects, have served as a source of inspiration for the model introduced in this study. Nevertheless, an important distinction needs to be emphasized. While in the case of insects, the objective is to exclusively represent a specific rotation, namely head direction, our aim is to extend the solution to encompass rotations of generic objects in a visual scene. This entails the necessity of combining the encoding of rotation with the identification of the object to which the rotation pertains.

In contrast to the ellipsoidal body's signal, which inherently implies that the rotation refers to the animal's body, our model requires visual identification of the object for which the rotation is being estimated. To accomplish this task, we rely on the well-established effectiveness of deep convolutional neural networks organized in an autoencoder model.

Deep convolutional networks have become the preferred choice for a wide range of applications, ranging from robotics (Bai et al., 2020; Ruiz-del-Solar and Loncomilla, 2020; Károly et al., 2021; Yu et al., 2023) to autonomous driving (Bojarski et al., 2017; Kuutti et al., 2019; Plebe et al., 2019a,b, 2024; Plebe and Da Lio, 2020). Convolutional networks overcome a long-standing challenge in computer vision: recognizing objects despite variations in their appearance, such as changes in illumination, size, and viewpoint. This property, known as *invariance*, is notably achieved with great proficiency in animals. However, the specific mechanisms employed by the brain to achieve invariance have been the subject of a long-standing debate that remains far from being resolved (Harris and Dux, 2005). Deep convolutional networks demonstrate an impressive level of invariance to object viewpoints, surpassing any previous method. Translation invariance is naturally achieved in the lower levels of a convolutional hierarchy due to the inherent nature of the convolution operation. On the other hand, rotation invariance becomes increasingly better in the higher layers and can be further improved by techniques such as data augmentation (Chen et al., 2020). However, while rotation invariance is advantageous for object recognition in many scenarios (Quiroga et al., 2023), there are situations where rotation sensitivity can be equally or even more beneficial.

Precise measurement of object rotation angles is crucial for a wide array of applications, for example estimating the pose of a vehicle obstacle in driving tasks. As mentioned above, most of these methods rely on deep neural networks. However, there is a contradiction here: these methods use neural networks that are optimized for rotation invariance to estimate rotations of objects. This may be inefficient in applications where rotation sensitivity is essential. Furthermore, these methods learn the rotation transformation indirectly from scratch rather than being explicitly designed to handle rotations.

Taking robotic grasping as an example, deep learning approaches generally employ a convolutional model followed by fully connected layers. The convolutional model is typically trained for a generic computer vision task, such as classification, while the final dense layers are fine-tuned for the specific pose estimation task. For example, in the model of Pinto and Gupta (2016), the last convolutional layer is followed by a dense layer of 18 units, which express the graspability scores for each angle in [0…180] degrees in steps of 10°. Therefore, the model is trained to solve an 18-way binary classification problem. The model in Viereck et al. (2017) is based on a network originally designed for handwritten digit classification. The model is a low-resolution convolutional network that learns a distance function between an action described as (*x, y*, θ) and a set of predefined viable grasp poses. In Mahler et al. (2017), a model called the Grasp Quality Convolutional Neural Network (GQ-CNN) predicts the success probability of grasps from depth images, where grasps are specified, once again, as (*x, y*, θ). Another study (Chen and Guhl, 2018) uses a standard convolutional model called Faster-R-CNN (Ren et al., 2016) to derive a bounding box of the object without explicitly measuring a rotation angle. The Faster-R-CNN uses a VGG-net as a backbone model and is trained on the ImageNet classification dataset. The work of Chen et al. (2022) applies a convolutional network called Panoptic FPN (Kirillov et al., 2018) to classify the object and match it with a set of known objects with preplanned grasping actions. The selected grasp is then transferred to the novel objects using geometric transformations.

It is not impossible to extract information about object rotation using convolutional networks. However, a challenge arises when using networks that are designed and trained for classification, as they are optimized for rotation invariance. These networks may not be able to generalize to new orientations effectively or may require extensive training data or augmentation techniques to learn how rotation operates.

We argue that our approach introduces a novel and more suitable method of encoding rotation, employing a cyclic structure that aligns with the cyclic nature of the rotation, similar to the ellipsoid body of insects or the r1π ring found in zebrafish larvae. The key distinction lies in the fact that, rather than shifting a single signal with the angle, our method involves shifting the entire vector that contains the latent representation of the object.

## 4 Model implementation

Our model aims to estimate the angle of rotation^1^ between two views of the same object. We adopt the classical autoencoder framework and create two networks: a “forward network” and an “inverse network”. The forward network learns to predict rotations by encoding an object's view and decoding a rotated version of it, while the inverse network estimates rotations by applying the trained encoder to two different views and predicting the angle of rotation between them.

First, let us define the operator that manipulates the latent space in such a way that the latent space retains the object's features regardless of the transformations applied to it. The *shift operator* is a matrix S_*n*_ of dimensions *R*×*R* that corresponds to a shift of *n* positions in a *R*-dimensional vector. The matrix is defined as follows:


(1) $$\begin{array}{l} S_{n}={\left[\begin{array}{ccccc} 0 & 0 & \cdots & 0 & 1\\ 1 & 0 & \cdots & 0 & 0\\ 0 & 1 & \cdots & 0 & 0\\ \ddots \\ 0 & 0 & \cdots & 1 & 0 \end{array}\right]}^{n}, \end{array}$$

with the convention that S_0_ is the identity matrix. It has been formally demonstrated that this kind of operator is linearly isomorphic to rotation (Serre and Scott, 1996; Bouchacourt et al., 2021). It is easy to see that, for a vector **v**, it holds **v** = S_*R*_**v**. In other words, the shift operator affects the latent vector in a cyclic way while preserving its information. That is what makes the latent space in our model “circular”.

The forward network takes two inputs: an image **x** of the object, an integer *r*∈[0…*R*) that expresses an angle of rotation α in a quantization of 2π into *R* intervals. We have the following:


(2) $$\begin{array}{l} \alpha =\frac{2\pi r}{R}. \end{array}$$

**Figure 2:**
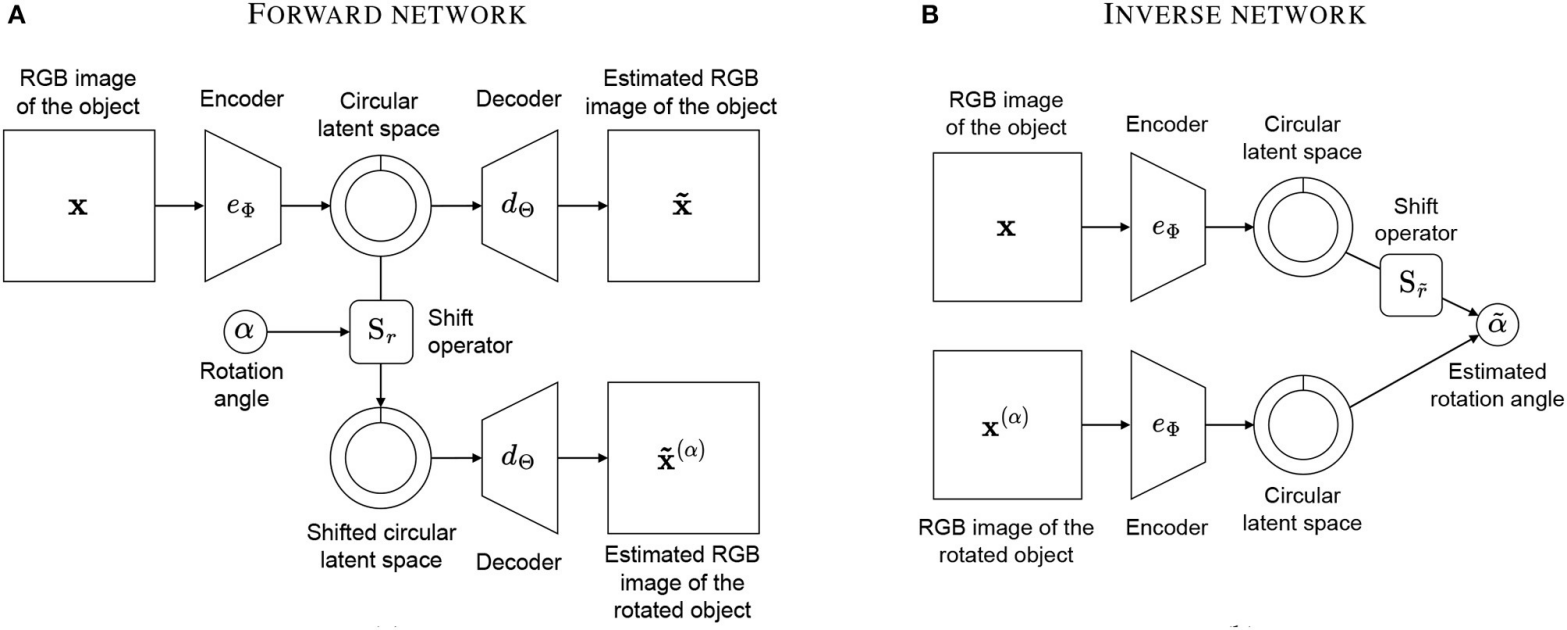
Our model consists of a forward network and an inverse network. The forward network **(A)** takes an image of an object and an integer representing an angle of rotation and produces an image of the object rotated by that angle as well as the original image. The inverse network **(B)** takes two images of an object with different rotations and estimates the angle of rotation re-using the encoders of the first network.

The network contains an encoder *e*_Φ_ with parameters Φ that compresses the high-dimensional input **x** into a latent representation **z** of dimension *R*. The low-dimensional representation **z** is fed to a decoder *d*_Θ_ with parameters Θ that learns to reconstruct the image **x**. In addition, the shift operator S_*r*_ is applied to **z** to shift the vector of *r* positions. The shifted latent space is fed to a second decoder that is the same instance of *d*_Θ_, and it learns to predict the image **x**^(α)^ of the object rotated by the angle α. The forward network is depicted in Figure 2A and is described by the following function:


(3) $$\begin{array}{l} f_{\Phi ,\Theta }\left(\text{x},r\right)=\left[\begin{array}{c} \tilde{\text{x}}\\ {\tilde{\text{x}}}^{\left(\alpha \right)} \end{array}\right], \end{array}$$

where:


(4) $$\begin{array}{lll} \tilde{\text{x}} & = & d_{\Theta }\left(e_{\Phi }\left(\text{x}\right)\right), \end{array}$$

(5) $$\begin{array}{lll} {\tilde{\text{x}}}^{\left(\alpha \right)} & = & d_{\Theta }\left({\text{S}}_{r}e_{\Phi }\left(\text{x}\right)\right). \end{array}$$

The loss function to train the forward model is the weighted sum of the mean squared errors of the reconstruction of the two images of the object, one in the original pose and the other with the object rotated by the angle α:


(6) $$\begin{array}{l} L_{\Theta ,\Phi }=\frac{1}{N}\overset{N}{\underset{i=1}{\sum }}\left(\lambda_{1}{\left({\text{x}}_{i}-{\tilde{\text{x}}}_{i}\right)}^{2}+\lambda_{2}{\left({\text{x}}_{i}^{\left(\alpha \right)}-{\tilde{\text{x}}}_{i}^{\left(\alpha \right)}\right)}^{2}\right). \end{array}$$

The inverse network consists of two instances of the encoder *e*_Φ_, with the same weights Φ obtained after training the forward network. Thus, all parameters of the inverse network are fixed and are not trained again. The two inputs of the network are an image **x** of the object and an image **x**^(α)^ of the object rotated by an angle α. The inverse network estimates the value of *r*∈[0…*R*]⊂ℕ so that $e_{\Phi }\left({\text{x}}^{\left(\alpha \right)}\right)={\text{S}}_{r}e_{\Phi }\left(\text{x}\right)$. This is equivalent to saying that the network “imagines” rotating the object in *e*_Φ_(**x**) until it finds the angle of rotation that best matches the rotation in $e_{\Phi }\left({\text{x}}^{\left(\alpha \right)}\right)$. The inverse network is depicted in Figure 2B and is defined by the following function measuring the cosine similarity between the latent vectors:


(7) $$\begin{array}{l} g_{\Phi }\left(\text{x},{\text{x}}^{\left(\alpha \right)}\right)=\underset{\tilde{r}}{\mathrm{argmin}}\left\{\frac{e_{\Phi }\left({\text{x}}^{\left(\alpha \right)}\right)\cdot \left({\text{S}}_{\tilde{r}}e_{\Phi }\left(\text{x}\right)\right)}{\left||e_{\Phi }\left({\text{x}}^{\left(\alpha \right)}\right)||\left||{\text{S}}_{\tilde{r}}e_{\Phi }\left(\text{x}\right)\right|\right|}\right\}. \end{array}$$

The network output $\tilde{r}$ can be transformed into a rotation angle using Equation (2). As mentioned above, the inverse network derives directly from the forward network. It does not have additional layers and it does not goes through any training or fine-tuning to estimate the rotation angle.

The inverse network can also be used to estimate the rotation of a known object from a single image. One way to do this is by creating a dataset of known objects, where each object is associated with a “default pose” represented by a unique circular latent vector. In this scenario, a single encoder *e*_Φ_ can be used to map the input image to the circular latent space, and the network can then utilize the latent vector of the memorized object to estimate the new pose.

In a famous work from 1971 Shepard and Metzler (1971), showed that the time required by humans to recognize that two images portray different orientations of the same 3D object is directly proportional to the angular difference in the orientations. It might be of interest to verify something similar in our artificial model. In its current design, however, it is not possible, as Equation (7) computes the argmin across all possible rotations of the object, hence taking the same computational time in every case. In a potential future implementation, we might consider employing heuristics to optimize the search for the minimum, thereby making our model more closely resemble this distinctive aspect of human perception.

### 4.1 Benchmark models

To evaluate the performance of our model, we implement three benchmarks with well-established architectures. Specifically, we used three vision networks with pre-trained weights that are available in the TensorFlow's model zoo: InceptionV3 (Szegedy et al., 2016), ResNet50 (He et al., 2016), and EfficientNetB4 (Tan and Le, 2019).

We train each of these models to directly predict the rotation angle α given the images **x** and **x**^(α)^, using a mean squared error loss function to compare the predicted angle with the original angle α. To achieve this, we duplicated the pre-trained encoder to produce two latent representations, one for each image and added two fully connected layers on top. The last layer of these models consists of two neurons trained to represent the values sinα and cosα. We train the models using transfer learning, thus freezing the pre-trained weights and training only the last two layers.

## 5 Datasets

We employ two datasets to evaluate the performance of our model. The first dataset encompasses only rotations in the image plane, which we used during the initial implementation phase. The second dataset incorporates rotations in 3D space from different viewpoints and is used in the comparison with the benchmark models.

### 5.1 COIL-proc dataset

The first dataset used in this study is a simplified version of the *Columbia University Image Library* also known as the COIL-100 dataset, which was originally presented in 1996 (Nene et al., 1996). COIL-100 is a collection of color images of 100 objects featuring small household items and toys. The objects are positioned on a motorized turntable against a black background and are captured from a fixed camera while the turntable rotates 360 degrees. This produces 72 poses for each object with a rotation step of 5 degrees.

**Figure 3:**
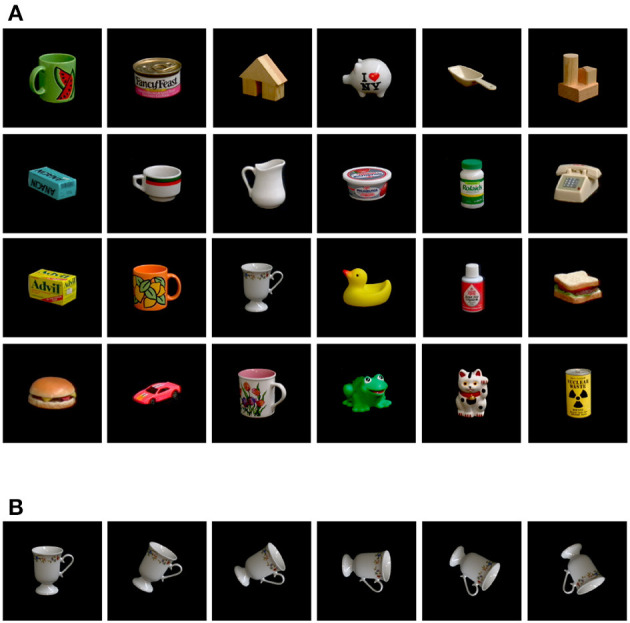
Our custom COIL-proc dataset derived from COIL-100 (Nene et al., 1996). **(A)** The selected 24 objects composing the dataset. **(B)** Sample of 2D rotation poses with steps of 30 degrees.

To test the model's performance on a basic rotation estimation task, we create a simplified dataset, which we called COIL-proc, by modifying COIL-100. Firstly, we reduce the number of objects from 100 to 24 by removing objects with shapes that were not significant to the task, such as round and uniform-shaped foods (e.g., fruits and donuts). We also excluded objects that are repeated with the same shape but in different colors, such as bottles, cans, and mugs. The 24 selected objects are shown in Figure 3A.

In COIL-proc, we generate the objects' poses in a different manner. Unlike COIL-100, where objects rotate in 3D along the vertical axis and their appearance changes significantly depending on the pose, we rotate the objects in 2D on the image plane as shown in Figure 3B. For each object, we select one “canonical pose” from the 72 available poses in which it is possible to appreciate the overall geometry of the object (for example, in the case of a mug, we select a pose where the handle is visible). From the canonical pose, we generate 71 additional poses by rotating the entire image by 5-degree steps, with the pivot point being the center of the image. We also apply padding to the images to ensure that the rotated poses fit within the canvas.

The resulting dataset contains 1,728 color images of 128 × 128 pixels. We split the data into a training set of 18 objects (1,296 images) and a test set of six objects (432 images). We consider four split combinations where we sample six different test objects each time. This is equal to performing a *k*-fold cross-validation with *k* = 4.

### 5.2 APC-proc dataset

**Figure 4:**
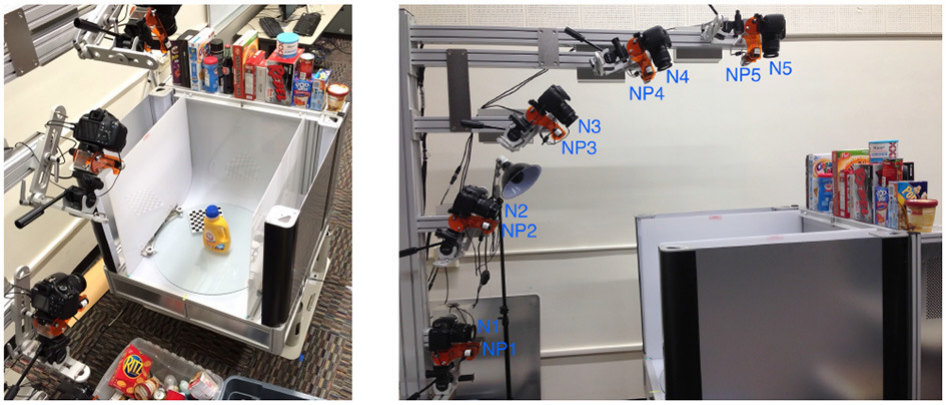
The data-collection process of the original APC dataset (Singh et al., 2014). On the left, a view of the desktop photography studio, with an object and the chessboard placed on the turntable. On the right, a side view of the five cameras (N1–N5) arranged in a quarter-circular arc.

The second dataset we use is a processed version of the *Amazon Picking Challenge Object Scans*, or APC dataset, which is an update to the previous BigBIRD dataset (Singh et al., 2014) modified to include the objects used in the Amazon Picking Challenge launched in 2015 (Correll et al., 2016). The dataset comprises images and point clouds for 27 items commonly sold on Amazon. The data is collected using a desktop photography studio (Figure 4, left) and five cameras arranged in a quarter-circular arc (Figure 4, right). The cameras are labeled N1 to N5, starting from the lowest position to the one directly above the turntable. The objects are placed on the turntable of the photography studio along with a chessboard necessary to obtain calibrated data. The turntable rotates in steps of 3 degrees, yielding 120 poses for each camera and a total of 600 images per object.

**Figure 5:**
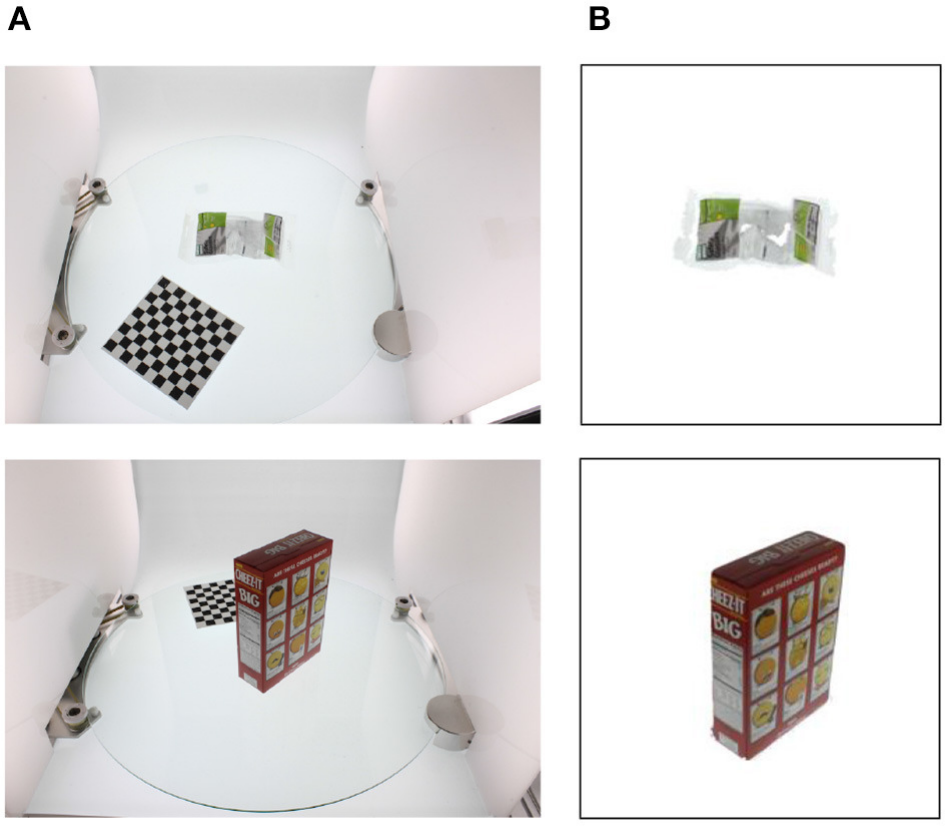
Results of our post-process on the APC dataset (Singh et al., 2014). **(A)** Original images in the APC dataset. **(B)** Corresponding processed images in our APC-proc dataset.

The APC dataset cannot be used as is in our application. In almost every image, the calibration chessboard is visible and rotates in exactly the same manner as the object. With this information, it would be impossible to determine if the model is learning the poses of the object or the pattern orientation of the chessboard. Therefore, it is essential to preprocess the dataset and remove the chessboard from the images. A simple crop operation would not suffice since the placement of the chessboard varies for each object, and in the case of cameras N1, N2, and N3, the object may occlude a part of the chessboard—see examples of Figure 5A. Fortunately, in addition to the point clouds, the APC dataset provides image segmentation masks generated from the object models. We use these masks to erase all pixels not belonging to an object and then crop the images to maximize the size of the object while ensuring that the poses remain within the image canvas. Figure 5B shows examples of processed images.

**Figure 6:**
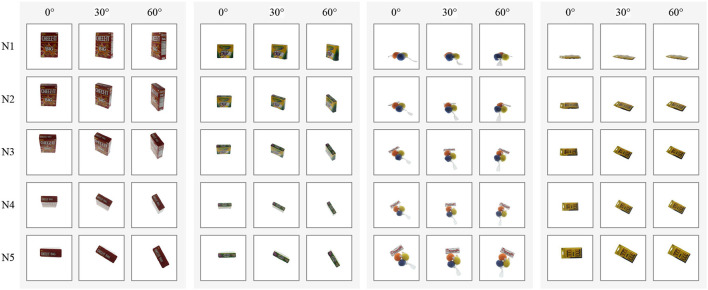
Samples of four objects in our custom APC-proc dataset derived from the Amazon Picking Challenge object scans (Singh et al., 2014). Rows show the point of views of the five cameras from N1 to N5; columns show three poses by steps of 30 degrees.

The resulting dataset comprises 16,200 color images of 128 × 128 pixels, with 20 objects allocated to the training set and 7 to the test set. To account for the different camera viewpoints, we split the dataset into five subsets corresponding to cameras N1–N5, and we train a different model for each subset. Figure 6 shows sample images for four objects arranged by camera viewpoints (rows), and object poses (columns). It is worth noting that the objects in the APC dataset are often positioned slightly away from the center of the turntable, resulting in a significant variation in their horizontal and vertical positions in the images. This differs from COIL-proc, where the objects are always centered in the image.

## 6 Results

Here we present the results of our models evaluated in the two datasets described in Section 5. For the second dataset, we also include a comparison with three benchmark vision models, and we show that our approach have competitive results and, in some cases, significantly surpass the baselines.

### 6.1 Results on the COIL-proc dataset

We evaluate our first model on the COIL-proc dataset, introduced in Section 5. As described in Section 4, the model is composed of a forward network and an inverse network. Only the forward network goes through training for 500 epochs. The inverse network, instead, derives the weights from the forward network and uses them to predict rotation angles. The encoder in both networks consists of two convolutional layers alternated with max-pooling layers, followed by three dense layers. The decoder in the forward network consists of three dense layers followed by three deconvolutional layers. The forward network has 3.8 million of trainable parameters, and the loss function is in Equation (6) with λ_1_ = λ_2_ = 0.5. The circular latent space is made up of 72 neurons; in other words, *R* = 72 in Equation (2). With 72 neurons, it is possible to represent rotations with a granularity of 5 degrees, which is the rotation step used in COIL-proc (and COIL-100).

To train the forward network, the dataset is organized into couples of input (**x**, *r*) and target (**x**, **x**^(α)^), as for Equation (3). Hence, there are 72^2^ possible combinations of inputs per object. To speed up training, we decide to reduce the number of samples by a factor of $\frac{1}{4}$. Specifically, for each of the 72 poses of **x**, the value of *r* spans the range [0…*R*] by steps of 4 (equal to 20 degrees). Therefore, the training set is made up of 23,328 samples, and the test set of 7,776 samples.

We split the training set and the test set based on the objects, as mentioned in §Section 5. In this way, the test set contains only objects that have never been seen during training. In addition, we perform a *k*-fold cross-validation with *k* = 4. In the end, the error estimation can be averaged over all *k* iterations to get the total effectiveness of the model.

Table 1 shows the results of the model on COIL-proc. The rows correspond to the four iterations of the *k*-fold cross-validation and the overall average. The four columns on the left report the performance of the forward network, whereas the two columns on the right report the performance of the inverse network. The forward network produces two outputs: the reconstruction of the original image **x**, and the prediction of the rotated image **x**^(α)^. For each image, we measure the mean squared error (MSE) (Bishop and Nasrabadi, 2006) and the structural similarity index (SSIM) (Wang et al., 2004). MSE values are in the range [0…+∞], where 0 indicates perfect similarity. SSIM values are in the range [−1…1], where 1 indicates perfect similarity, 0 indicates no similarity, and −1 indicates perfect anticorrelation.

**Table 1:** Results of our model on the COIL-proc dataset.

	**Original image**		**Rotated image**		**Rotation angle**	
	**MSE ↓**	**SSIM ↑**	**MSE ↓**	**SSIM ↑**	***E*2π ↓**	***Eπ* ↓**
Iter 1	0.0178	0.8389	0.0178	0.8391	0.0536	0.0457
Iter 2	0.0173	0.8367	0.0171	0.8369	0.0922	0.0494
Iter 3	0.0081	0.8419	0.0085	0.8352	0.0120	0.0315
Iter 4	0.0121	0.8224	0.0128	0.8142	0.0163	0.0722
Mean	0.0144	0.8392	0.0145	0.8371	0.0526	0.0422

**Figure 7:**
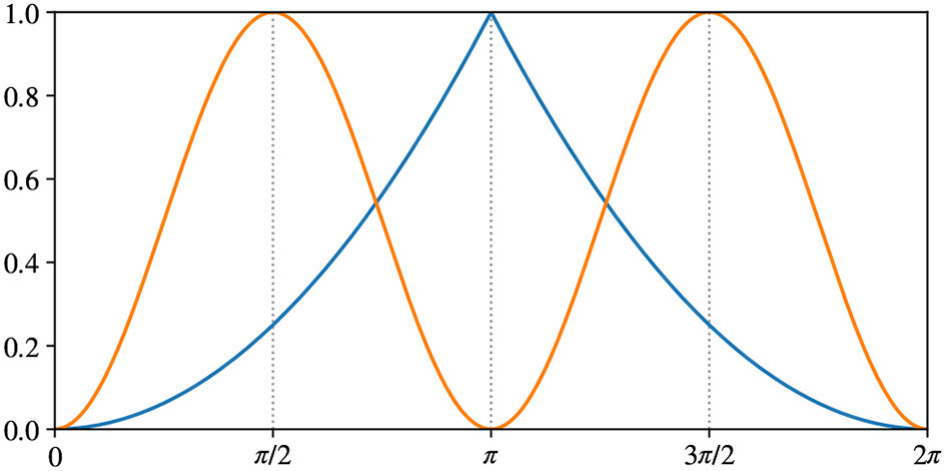
Difference between our metrics measuring rotation errors: in blue *E*2π from Equation (8), in orange *Eπ* from Equation (9).

The output of the inverse network is the estimation $\tilde{\alpha }$ of the rotation angle α between the two poses of the object passed as input images (**x**, **x**^(α)^). We define the following two error measures:


(8) $$\begin{array}{lll} E2\pi & = & {\left(\frac{1}{\pi }atan2\left(\sin\left(\alpha -\tilde{\alpha }\right),\cos\left(\alpha -\tilde{\alpha }\right)\right)\right)}^{2} \end{array}$$

(9) $$\begin{array}{lll} E\pi & = & \sin^{2}\left(\alpha -\tilde{\alpha }\right) \end{array}$$

where atan2 is the 2-argument arctangent. Figure 7 shows the difference between the two error measures. Both errors are in range [0…1], but *E*2π has maximum at $\alpha -\tilde{\alpha }=\pi$, while *Eπ* has maximum at $\alpha -\tilde{\alpha }=\frac{\pi }{2}+k\pi$. The choice of *E*2π is straightforward: π is the maximum possible difference in angle. Hence, it corresponds to the maximum error. We add *Eπ* to better evaluate the model in the context of a pick-and-place application because the grasping pose is usually equivalent when rotated 180°. We will further discuss this point when presenting the results on APC-proc in Section 5.2.

The results in Table 1 appear to be consistent across the iterations of the cross-validation. Different train/test splits yield similar values for all metrics. Furthermore, the reconstruction error of the rotated image **x**^(α)^ (columns 3 and 4) remains very close to the error of the original image **x** (columns 1 and 2), for both metrics MSE and SSIM. Lastly, the average values of *E*2π and *Eπ* are similar and correspond to approximately half shift in the 72 neurons' discretization. However, the metric *Eπ* will be more meaningful in the APC-proc, as we will see later in this Section.

**Figure 8:**
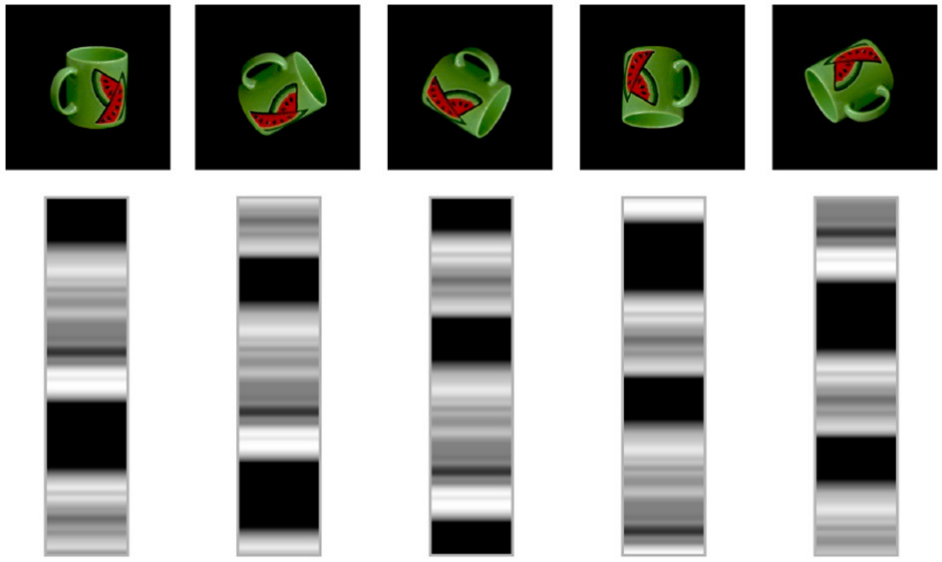
Visualization of a circular latent space learned by our model on the COIL-proc dataset. In each column, the latent vector is shifted by 12 units, which equals to rotating the image by 60 degrees.

We include in Figure 8 a visualization of the latent space learned by the model. The figure shows five rotations in steps of 60 degrees and the related latent space of 72 neurons generated by our model. It is possible to appreciate the correspondence between the rotation in the image space and the shift operator in the circular latent space.

### 6.2 Results on the APC-proc dataset

To fully evaluate the performance of our model, we selected the APC-proc dataset. This dataset was chosen because it provides multiple views of objects from various angles and includes objects with complex geometries, including transparent objects. Furthermore, since most objects are displaced from the center of the turntable (as explained in Section 5.2), our model must be robust to translations with respect to the center of the image.

The APC-proc dataset—as well as the original APC dataset—is organized into five sub-datasets according to the five cameras used in the data collection process (Figure 4, right). The same rotations produce significantly different poses in each sub-dataset. For instance, images captured by camera N5, which is the top-most camera, have poses that resemble those in COIL-proc, as shown in the last row of Figure 6. On the other hand, the sub-dataset of camera N1, which is the front camera, yields entirely different poses that can lead to widely varying images depending on the shape of the objects, as evident from the disparity between the left-most and right-most images in the first row of Figure 6. Consequently, we opted to train five different models, one for each camera and its corresponding point of view.

Each of the five models consists of a forward network and an inverse network with the same structure presented in the previous Section 6. However, in this case, the circular latent space's size is *R* = 120 in Equation (2), since APC-proc has a granularity of 3 degrees, resulting in 120 poses per object. The forward network has 10 million trainable parameters and undergoes 100 epochs of training with the loss function specified in Equation (6). For each sub-dataset, we randomly choose 20 objects for the training set and 7 for the test set, ensuring that the model is always evaluated on objects that it has never seen before. Similar to COIL-proc, we reduce the number of samples to accelerate training, but this time by a factor of $\frac{1}{2}$. Eventually, each sub-dataset contains 144, 000 training samples and 50, 400 test samples.

Table 2 shows the results of the five models. The rows correspond to the models, each using one of the sub-datasets from N1 to N5. The four columns on the left report the performance of the forward network, and the two columns on the right report the performance of the inverse network. Similarly to the results on COIL-proc, for each output of the forward network, we measure the mean squared error and the structural similarity index. For the output of the inverse network, we use the two error measures defined in Equations (8) and (9). Despite APC-proc being a more challenging dataset, the models show similar results to the COIL-proc dataset. The MSE and SSIM scores are even better in this case. The only discrepancy is in the values of *E*2π and *Eπ*, which get significantly worse when using front-facing cameras, such as N1 and N2. This is no surprise: the point of view in N1 and N2 is virtually perpendicular to the rotation axis, and the consequent partial occlusion makes it harder to estimate the rotation. This is the reason why the error *Eπ* in the case of camera N1 is five times larger than in N5.

**Table 2:** Results of our models on the APC-proc dataset.

	**Original Image**		**Rotated Image**		**Rotation Angle**	
	**MSE** ↓	**SSIM** ↑	**MSE** ↓	**SSIM** ↑	*E*2π ↓	*Eπ* ↓
N1	0.0108	0.8900	0.0159	0.8804	0.2015	0.2719
N2	0.0091	0.9147	0.0124	0.9053	0.1732	0.2108
N3	0.0081	0.9137	0.0110	0.9057	0.1478	0.1416
N4	**0.0046**	**0.9392**	**0.0057**	**0.9336**	0.0771	0.0868
N5	0.0092	0.8961	0.0104	0.8909	**0.0544**	**0.0534**
Mean	0.0083	0.9107	0.0111	0.9031	0.1308	0.1529

The values in bold represent the best results as measured by the metrics in the respective columns.

We conclude with a comparison between our model and three benchmarks based on commonly adopted vision models: InceptionV3 (Szegedy et al., 2016), ResNet50 (He et al., 2016), and EfficientNetB4 (Tan and Le, 2019). Details about the implementation of the models are in Section 4.1. Recall that these models are trained to estimate the angle of rotation between two input images directly. Training takes 100 epochs, and the number of trainable parameters is 17 million for InceptionV3, 67 million for ResNet50, and 58 million for EfficientNetB4. As we do for our model, we train a separate model for each sub-dataset; moreover, for the sake of time, we discard the sub-datasets of cameras N2, N3, and N4, and we train only on N1 and N5.

The results of the comparison are given in Table 3. Our inverse model outperforms all the other three except for the value of *E*2π in N1, which is better for EfficientNetB4—the model that performs better among the three considered. According to the previous results in Table 2, all models have lower errors in camera N5 with respect to N1. However, this time it is possible to appreciate a discrepancy between the values of *E*2π and *Eπ*. In the benchmark models, *Eπ* is nearly three times higher than *E*2π. In our model, instead, the difference between *Eπ* and *E*2π is much lower. In the contest of many robotic applications, such as pick-and-place tasks, this discrepancy is meaningful. The *Eπ* metric does not penalize the model when it incorrectly estimates a rotation by 180 degrees because, for most objects, the final pose is the same—for example, the boxes in Figure 6 are almost perfectly symmetric. When performing pick-and-place, the grasping movement is the same if the object has rotation α or α+π. For this reason, it is more effective to evaluate the accuracy of the models with *Eπ* when considering potential robotic applications. When doing so, our model is shown to outperform all other models, despite having nearly six times fewer trainable parameters than EfficientNetB4 (the best of the three benchmark models). Moreover, recall that while the benchmark models are directly trained to evaluate the rotation between images, our model is trained to reconstruct image output, and the inverse network never goes through a training phase.

**Table 3:** Comparison between our model and three benchmark models on the APC-proc dataset.

	**Camera N1**		**Camera N5**	
	***E*2π ↓**	***Eπ* ↓**	***E*2π ↓**	***Eπ* ↓**
InceptionV3 (Szegedy et al., 2016)	0.2858	0.4707	0.1863	0.4306
ResNet50 (He et al., 2016)	0.2101	0.4523	0.0912	0.2452
EfficientNetB4 (Tan and Le, 2019)	**0.1664**	0.4104	0.0617	0.2043
Ours	0.2015	**0.2719**	**0.0544**	**0.0534**

The values in bold represent the best results as measured by the metrics in the respective columns.

### 6.3 Analysis of the circular latent space

To conclude, we conduct an analysis of the structure within the circular latent space learned by the presented model. We focus on the model trained on the COIL-proc dataset due to the limited number of objects and the reduced size of the latent space (72 neurons instead of 120 for the APC-proc), which facilitates a clearer visualization of the internal structure. We investigate whether the latent space, due to the well-documented polysemous nature of neurons in artificial (and biological) neural networks, demonstrates a superposition of coding related to rotations, irrespective of the object category, intertwined with coding related to the objects themselves.

**Figure 9:**
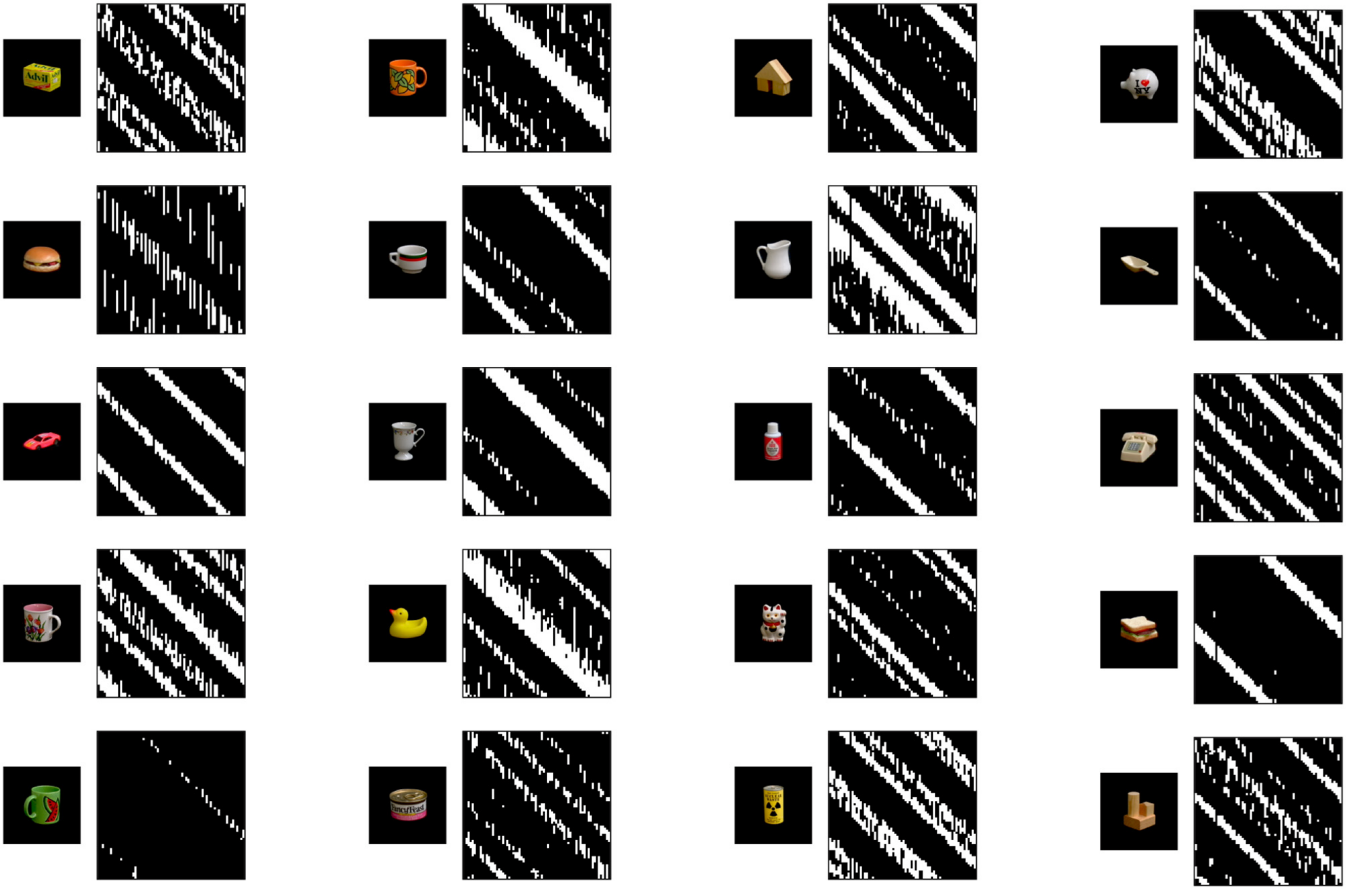
Visualization of neurons in the latent space that exhibit strong correlations with specific objects from the COIL dataset. The 72 possible rotations of each object are considered separately. Detailed description of the matrices in the text.

In a first analysis, which results are in Figure 9, we aim to identify neurons within the latent space that exhibit stronger correlations with specific objects from the COIL dataset. This analysis is performed considering the 72 possible rotations of each object separately.

Let *z*_*i*_(*r, o*) be the activation value of the *i*-th neuron in the latent space **z** encoding an image of the object *o*∈*O* with an index of rotation *r*∈[0…*R*), and *i*∈[0…*R*). For each object *o*, we compute a matrix *R*×*R*, where the number of rows represent all rotations *r*. Each row consists of an array of binary values *v*_*i*_(*r, o*) describing if the *i*-th neuron in the latent space is strongly associated with the object *o*. Specifically, being ō∈*O*\{*o*}, we define *v*_*i*_(*r, o*) as follows:


(10) $$v_{i}(r,o)=\{\begin{array}{ll} 1 & \text{if }z_{i}(r,o)>\mu \left(z_{i}(r,\bar{o})\right)+\sigma \left(z_{i}(r,\bar{o})\right)\\ 0 & \text{otherwise} \end{array},$$

where μ is the average and σ the standard deviation of the activation values over all possible ō.

The results in Figure 9 show how, for all objects, the shifting pattern of activation does not originate from a single distinct cluster of neurons. Furthermore, there is minimal overlap in the patterns for the same rotation across all objects. This implies that the neurons in the latent space are effectively distributed in their representation of objects, encoding various distinct features. Additionally, the consistent diagonal pattern in the matrices confirms that the model correctly maps rotations in the world with shifts within the circular latent space.

We extend our investigation to identify neurons in the latent space that primarily represent object identity, regardless of rotation. To do this, we follow a similar approach to our previous analysis, but this time, we consider all rotations collectively.

**Figure 10:**
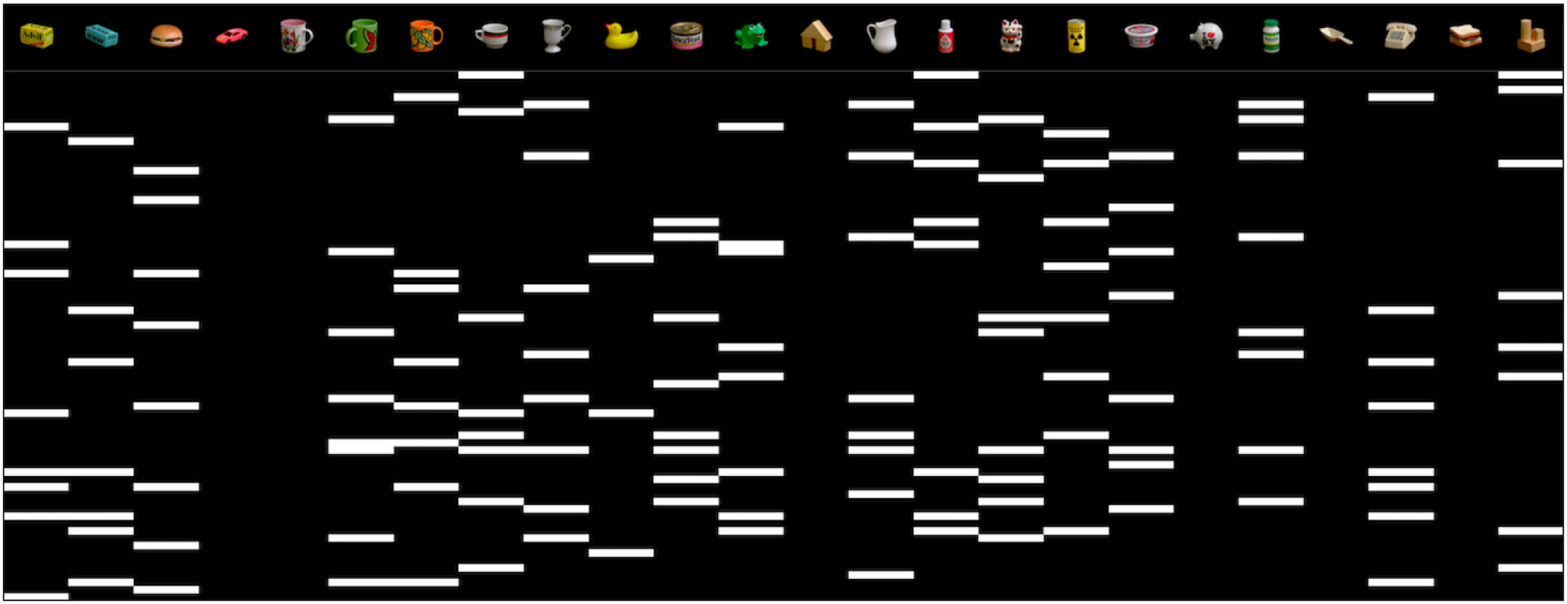
Visualization of neurons in the latent space that exhibit strong correlations with specific objects from the COIL dataset, mediated over all rotations. Detailed description of the matrix in the text.

Let *z*_*i*_(*o*) be the average activation value of the *i*-th neuron of **z** encoding the object *o* over all *R* rotations, with *i* ∈ [0…*R*). We define a score *s*_*i*_(*o*) of the *i*-th neuron over all rotations as follows:


(11) $$\begin{array}{l} s_{i}\left(o\right)=z_{i}\left(o\right)-\mu \left(z_{i}\left(ō\right)\right), \end{array}$$

where μ is the average over all possible ō. For each object *o*, we indicate the *M* neurons with the highest non-negative scores *s*_*i*_(*o*). In Figure 10, we used *M* = 8.

The results of this second analysis are reported in Figure 10. In contrast to the first analysis, in this case, the number of neurons involved in coding for individual objects is lower, as expected due to the constraint of maintaining the shifting property for rotation. For these select neurons, the results remain consistent with our previous analysis, revealing a uniform distribution of neurons across the various objects, even though it becomes more sparse when distilled for all rotations.

## 7 Conclusions

We have presented a model to estimate the difference in rotation between two views of the same object. Our model is explicitly designed to incorporate prior knowledge about the rotation operation, in a way that resembles the neural circuits used by *Drosophila*'s brain to represent head directions. In our model, the rotation transformation is encoded within the network as an operator that cyclically manipulates the internal latent space. The operator works by shifting the elements in the circular latent vector. When the vector is decoded, it produces an image of the object that is rotated relative to its original pose while maintaining its original features.

We have demonstrated the model's performance on two datasets that we pre-processed to be best suited for rotation estimation: COIL-proc and APC-proc. We have evaluated the results—both as image output and as rotation angles—using four different metrics, and the model performs effectively in all four. In addition, we have compared our model with three benchmarks based on widely employed vision models. Despite being a much smaller neural network, our model significantly outperforms the benchmarks in most cases.

As part of future work, we intend to address the main limitation of our model, which is its assumption of a single axis of rotation. This will likely require us to elaborate further on the structure of the circular latent space and how the shift operator manipulates it. Once the model is capable of processing multiple axes of rotation, it will have the potential to be integrated into practical robotic applications, such as pick-and-place tasks. Furthermore, because of its compact size, the model can also serve as a module in other complex pipelines that require rotation estimation, such as a stereo vision system. Furthermore, we will explore novel applications to showcase the usefulness of this concept, particularly in the domains of robotics and autonomous driving.

## Data availability statement

The raw data supporting the conclusions of this article will be made available by the authors, without undue reservation.

## Author contributions

AP: Conceptualization, Data curation, Investigation, Methodology, Software, Writing—original draft. MD: Conceptualization, Supervision, Writing—review & editing.

## Footnotes

^1^We assume a single axis of rotation. Extensions to multiple rotation axes are discussed in future work in Section 7.
